# Petrography, stable isotope compositions, microRaman spectroscopy, and presolar components of Roberts Massif 04133: A reduced CV3 carbonaceous chondrite

**DOI:** 10.1111/maps.12377

**Published:** 2014-11-07

**Authors:** Jemma Davidson, Devin L Schrader, Conel M O'D Alexander, Dante S Lauretta, Henner Busemann, Ian A Franchi, Richard C Greenwood, Harold C Connolly, Kenneth J Domanik, Alexander Verchovsky

**Affiliations:** 1 Lunar and Planetary Laboratory, University of Arizona1629 E. University Blvd., Tucson, Arizona, 85721–0092, USA; 2 Planetary and Space Sciences, The Open UniversityWalton Hall, Milton Keynes, Buckinghamshire, MK7 6AA, UK; 3 Department of Mineral Sciences, National Museum of Natural History, Smithsonian Institution, 10th & Constitution NWWashington, District of Columbia, 20560–0119, USA; 4 Department of Terrestrial Magnetism, Carnegie Institution of Washington5241 Broad Branch Road NW, Washington, District of Columbia, 20015–1305, USA; 5 School of Earth, Atmospheric and Environmental Sciences, The University of ManchesterOxford Road, Manchester, M13 9PL, UK; 6 Department of Physical Sciences, Kingsborough Community College of the City University of New York2001 Oriental Blvd., Brooklyn, New York, 100235, USA; 7 Earth and Environmental Sciences, The Graduate Center of the City University of New York365 5th Ave., New York City, New York, 10016, USA

## Abstract

Here, we report the mineralogy, petrography, C-N-O-stable isotope compositions, degree of disorder of organic matter, and abundances of presolar components of the chondrite Roberts Massif (RBT) 04133 using a coordinated, multitechnique approach. The results of this study are inconsistent with its initial classification as a Renazzo-like carbonaceous chondrite, and strongly support RBT 04133 being a brecciated, reduced petrologic type >3.3 Vigarano-like carbonaceous (CV) chondrite. RBT 04133 shows no evidence for aqueous alteration. However, it is mildly thermally altered (up to approximately 440 °C); which is apparent in its whole-rock C and N isotopic compositions, the degree of disorder of C in insoluble organic matter, low presolar grain abundances, minor element compositions of Fe,Ni metal, chromite compositions and morphologies, and the presence of unequilibrated silicates. Sulfides within type I chondrules from RBT 04133 appear to be pre-accretionary (i.e., did not form via aqueous alteration), providing further evidence that some sulfide minerals formed prior to accretion of the CV chondrite parent body. The thin section studied contains two reduced CV3 lithologies, one of which appears to be more thermally metamorphosed, indicating that RBT 04133, like several other CV chondrites, is a breccia and thus experienced impact processing. Linear foliation of chondrules was not observed implying that RBT 04133 did not experience high velocity impacts that could lead to extensive thermal metamorphism. Presolar silicates are still present in RBT 04133, although presolar SiC grain abundances are very low, indicating that the progressive destruction or modification of presolar SiC grains begins before presolar silicate grains are completely unidentifiable.

## Introduction

Roberts Massif (RBT) 04133 was found as a single stone weighing 459.4 g during the 2004 ANSMET field season at Roberts Massif, Antarctica. This Antarctic find was initially classified as a petrologic type 2 Renazzo-like carbonaceous (CR) chondrite with a B/C weathering grade (Weisberg et al. 2008). We report a multitechnique study of the petrography, stable isotope compositions, Raman spectral characteristics, and presolar components of RBT 04133. We present data that indicate RBT 04133 is a reduced Vigarano-like carbonaceous (CV3) chondrite.

In general, chondrites can be classified on the basis of their petrography and mineralogy (e.g., Weisberg et al. 2006), bulk element abundances (e.g., Kallemeyn et al. 1989), stable O-isotope compositions (e.g., Clayton and Mayeda 1999), and the C and N isotopic compositions of their insoluble organic matter (IOM; Alexander et al. 2007), although there is often some overlap between groups. The petrologic subtypes of type 3 (i.e., least altered) samples can be determined by various means, including: Cr_2_O_3_ content of FeO-rich olivine (for types 3.00 to 3.2; Grossman and Brearley 2005), Ni and Co content of metal (e.g., Kimura et al. 2008), and the Raman spectral parameters of IOM (e.g., Quirico et al. 2003; Bonal et al. 2006; Busemann et al. 2007).

The CV chondrites are divided into three subgroups on the basis of their petrography and mineralogy: the reduced Vigarano-like (CV3_red_), the oxidized Allende-like (CV3_oxA_), and the oxidized Bali-like (CV3_oxB_) chondrites (e.g., McSween 1977; Kimura and Ikeda 1997; Weisberg et al. 1997; Krot et al. 1998, 2000). The modal mineralogies of the CV3_red_ and CV3_ox_ subgroups are somewhat distinct (Howard et al. 2010). For example, CV3_ox_ chondrites contain between 1.9 and 4.2 vol% phyllosilicates, which are typically absent in CV3_red_ chondrites (Howard et al. 2010). Some samples are intermediate between subgroups (e.g., Mokoia contains both CV3_oxA_ and CV3_oxB_ lithologies; Krot et al. 1998) or contain clasts of material from other subgroups (e.g., Vigarano contains clasts of oxidized CV material; Krot et al. 2000). As a result, the CV3 chondrites are considered to be regolith breccias from a single parent body (Krot et al. 2000).

Raman spectroscopy is useful for determining the relative degree of disorder (or maturity) of organic matter in meteorites (e.g., Quirico et al. 2003; Bonal et al. 2006; Busemann et al. 2007), IDPs (e.g., Wopenka 1988; Quirico et al. 2005; Busemann et al. 2009; Davidson et al. 2012), and cometary samples (e.g., Rotundi et al. 2008; Busemann et al. 2009; Davidson et al. 2012). The Raman spectra of samples containing disordered carbonaceous material are dominated by two bands: the D (“disordered”; breathing mode only active in disordered carbon; e.g., Ferrari and Robertson 2000) and G (“graphite”) bands centered near 1360 and 1590 cm^−1^, respectively (Rotundi et al. 2008 and references therein). With increasing disorder, these bands broaden significantly and the apparent G-band position moves toward higher wavenumbers. The D- and G-band peak parameters (relative intensities [I], full widths at half maximum [Γ], and peak positions [ω]) detected in the organic matter are correlated with the alteration processes it experienced, such as parent body thermal metamorphism (e.g., Quirico et al. 2003; Bonal et al. 2006; Busemann et al. 2007). Raman spectroscopy can be used to estimate the peak metamorphic temperatures (PMTs) experienced by individual chondrites in their parent body (Busemann et al. 2007). The PMTs for the CV3 chondrites range from 260 to 590 °C (Busemann et al. 2007), similar to those determined by X-ray absorption near edge structure (XANES) spectroscopy of IOM (415–554 °C; Cody et al. 2008). Typically, IOM from the CV3_red_ chondrites experienced lower temperatures than that from the CV3_ox_ subgroups (Busemann et al. 2007; Cody et al. 2008). This demonstrates that the members of the CV chondrite group have experienced variable degrees of thermal metamorphism.

The types and abundances of presolar grains (microscopic dust grains formed in the outflows and cooling gases of stars prior to the formation of our solar system; Zinner 2014) present in a sample can also be used to qualitatively investigate its alteration history as they are typically found in high abundance in the least altered chondrites (e.g., Nguyen et al. 2010) and in lower abundance, in more aqueously or thermally altered samples (e.g., Huss and Lewis 1995; Davidson et al. 2014a). Presolar grain types include nanodiamonds, silicon carbide (SiC), graphite, oxides, and silicates; their resilience to parent body alteration depends upon grain type, with silicates generally being the most susceptible to destruction (Zinner 2014).

## Experimental Methods

### Samples and Sample Preparation

A chip of RBT 04133 (split 5 from parent 3; 2.3 g) from the interior of the meteorite was obtained to avoid fusion crust and heavily terrestrially weathered portions of the whole stone. A thin section of the meteorite (RBT 04133,8) was also obtained. Crushed and homogenized whole-rock material (a 200 mg aliquot from 2 g of powdered sample) was used for O, C, and N stable isotope analyses. A sample was also leached with HCl to estimate the degree of terrestrial weathering (e.g., Bland et al. 2000; Greenwood et al. 2008) at the Open University (OU). An IOM residue (3 mg) was prepared using 1 g of material following the method of Cody and Alexander (2005) at the Carnegie Institution of Washington (CIW). The IOM residue was used for C and N isotopic analysis, Raman spectroscopy, and previously reported NanoSIMS ion imaging (Davidson et al. 2010, 2014a). Matrix material was extracted from crushed whole-rock material for NanoSIMS ion imaging; fragments were individually pressed into high-purity Au foils mounted on 10 mm Al stubs (see Davidson 2009 and Davidson et al. [2014a] for detailed methodology).

### Light Element Stable Isotope Analysis

Whole-rock O-isotope analyses were undertaken at the OU on unwashed, homogenized samples (from a 200 mg split of 2 g of powdered whole-rock sample) and a leached subsample (see Greenwood et al. 2008) using an infrared laser fluorination system (Miller et al. 1999). Prior to laser fluorination, the system blank was reduced by flushing the chamber with aliquots of BrF_5_. The O_2_ released by laser fluorination was purified by two cold traps on either side of a KBr bed (heated to 110 °C). A Micromass Prism III dual inlet mass spectrometer analyzed the isotopic composition of the O_2_. Interference at *m*/*z* = 33 by NF^+^ was monitored by performing scans for NF_2_^+^ on the sample gas before analysis, and was below detection limits at all times. Analytical precision (1σ) for homogeneous samples, based on replicate analyses of international (NBS-28 quartz, UWG-2 garnet) and internal standards, was approximately ±0.04‰ for δ^17^O, ±0.08‰ for δ^18^O, and ±0.024‰ for Δ^17^O (Miller et al. 1999). Isotopic compositions are reported relative to standard mean ocean water and Δ^17^O was calculated as: Δ^17^O = δ^17^O − 0.52 × δ^18^O.

Carbon and N stable isotope analyses were undertaken at the OU on powdered and homogenized whole-rock subsamples (2.036 mg) and IOM residues (0.332 mg) using the *Finesse* mass spectrometer system (e.g., Verchovsky et al. 1997). The *Finesse* system consists of several mass spectrometers connected to a single extraction system to allow simultaneous isotope and element analysis of N and C. Stepped pyrolysis, combustion, or a combination of the two techniques, at temperatures up to 1450 °C, was used to analyze microgram-sized samples. A high-sensitivity capacitance manometer allowed for the precise measurement of the amount of C in the form of CO_2_ (Verchovsky et al. 1997).

### MicroRaman Spectroscopy

Laser Raman analyses of IOM were conducted with a Horiba Jobin Yvon Labram HR Raman system at the OU (see also Rotundi et al. 2008). Excitation was delivered by an argon ion laser (514.5 nm) and the spectra were acquired with a spectral resolution of 3 Δcm^−1^ using a 600 g mm^−1^ grating. The laser delivered a power of 0.07 mW at the sample surface. The beam was focused with a ×100 long working distance objective giving a spatial resolution of approximately 1.2 μm. Spectra were recorded across the whole of each particle with a 1.2 μm step in both *x* and *y* directions. Spectra were accumulated as 5 sets of 30 s integrations for a total analysis time of 150 s per spot. A total of 150 spectra were accumulated over three large (each approximately 50 × 50 μm in diameter) IOM fragments (84, 34, 32 spectra each). The carbonaceous D- and G-band features (intensity, position, and full width at half maximum) were fitted in the spectral range of 850–2100 cm^−1^ to Lorentzian profiles. The steep sloping fluorescence baseline was subtracted with a free-floating linear background. Following the method of Busemann et al. (2007) only spectra that fit strict criteria were used for further data reduction. Spectra were excluded if they exhibited a large fluorescence background; large relative fitting errors (>100%) for band areas, widths, or positions; or band widths/positions that were more than 3σ from the average. The reported parameters are averages of all spectra that meet the selection criteria (145 of 150 spectra; see Busemann et al. 2007 for detailed selection criteria and data reduction procedures).

### NanoSIMS Ion Imaging

Raster ion images of 15 matrix fragments (15–25 μm in diameter; total area = 2700 μm^2^) were collected using the Cameca NanoSIMS 50L at the OU. The fragments were presputtered with a 16 keV Cs^+^ primary ion beam and currents of typically 100 pA for 5–10 min (depending on fragment size) until sputter equilibrium was reached. All analyses were undertaken with currents of 1 pA and raster sizes of 512 × 512 pixels (for areas of 15 × 15 to 25 × 25 μm^2^), providing a typical spatial resolution of better than 150 nm.

Two sets of measurements were performed on the same areas: (1) to locate C-anomalous phases such as presolar SiC and other C-/N-anomalies (^12^C^−^, ^13^C^−^, ^12^C^14^N^−^, ^12^C^15^N^−^, ^16^O^−^, ^28^Si^−^, and ^24^Mg^16^O^−^; 70 min total analysis time), and (2) to locate O anomalous phases such as presolar oxides and silicates (^16^O^−^, ^17^O^−^, ^18^O^−^, ^28^Si^−^, ^29^Si^−^, ^30^Si^−^, and ^24^Mg^16^O^−^; 240 min total analysis time; Davidson et al. 2010). A mass resolution of *m/*Δ*m* = 9000 (Cameca definition) was used to resolve interferences from neighboring peaks (e.g., ^10^B^16^O^−^ from ^12^C^14^N^−^ and ^11^B^16^O from ^12^C^15^N^−^ for the first set of measurements; ^17^O^−^ from ^16^OH^−^ in the second set of measurements). The isotopes ^28^Si and ^24^Mg^16^O were measured to distinguish between silicate and oxide grains.

Ion images were processed and quantitatively analyzed with the L'IMAGE software (L. R. Nittler, CIW). Prior to data extraction, individual image planes were aligned with each other to correct for stage and/or beam drift during the measurement. See Davidson (2009) for complete measurement conditions and data reduction procedures. The presolar SiC abundance determined by NanoSIMS raster ion imaging of RBT 04133 IOM was previously reported by Davidson et al. (2014a).

### Mineralogy and Petrography Analysis

An optical microscope was used to initially characterize the thin section. Backscattered electron (BSE) and X-ray element maps were obtained (operating conditions: 15.0 kV and 40.0 nA) with the Cameca SX-50 electron probe microanalyzer (EPMA) at The University of Arizona's Lunar and Planetary Laboratory (LPL). These maps show the elemental and mineralogical distributions within the sample, and were used to identify mineral phases for study. High-resolution BSE and secondary electron images were obtained for each chondrule selected for study using a dual beam FEI Quanta 3-D scanning electron microscope (SEM) at the OU. Modal abundances of different phases (Table 1) were determined from BSE images of the full thin section and of individual chondrules using the IQmaterials® program and pixel (i.e., point) counting in Adobe Photoshop® (e.g., Schrader et al. 2011; N.B., area% determined by point counting is equivalent to vol%; Eisenhour 1996 and references therein). Apparent chondrule diameters were determined by measuring the major and minor axes of chondrules in BSE images using Adobe Photoshop®.

**Table 1:** Modal abundances (vol%) of mineralogical components within RBT 04133,8 compared to other carbonaceous chondrite groups

Component	RBT 04133^a^	CV^b^	CK^b^	CR^c^	CO^b^	CM^b^	CI^b^
Opaques (metal + sulfide + oxide)	7	0–5	0–5	10	1–5	0.1	<<1
Refractory inclusions (CAIs +AOAs)	8	10	10	0.5	13	5	<<1
Chondrules	37	45	45	65	48	20^d^	<<1
Type I	90	95^e^		96			
Type II	6	5^e^		4			
Al-rich	4						
Matrix + dark inclusions	55	40	40	35	34	70^d^	>99

a Modal abundances do not add up to 100% as opaque minerals are present within matrix and chondrules. Estimated errors are approximately 10% of the reported values.

b Data from Weisberg et al. (2006) and references therein.

c Schrader et al. (2011).

d Highly variable.

e Jones (2012).

Major and minor element abundances (Na, Si, Mg, Al, P, Ca, K, Mn, Ti, Fe, Cr, Ni, and Zn for silicate phases; Na, Si, Mg, Al, P, S, Ca, Cr, Mn, Ti, Fe, Ni, Co, Cu, and Zn for opaque phases) were determined quantitatively with the Cameca SX-50 EPMA at the LPL. The polished thin section was carbon-coated and analyzed with a 1 μm beam (operating conditions: 15 keV and 20 nA), a PAP correction method (a Phi-Rho-Z correction technique; Armstrong 1988), and counting times of 20 s on the peak and 10 s on each background for a total of 40 s per element. Standards (with detection limits in wt%) are listed in Table 2 for silicate analyses and Table 3 for metal and sulfide analyses. Only metal and sulfide, and stoichiometric olivine and pyroxene analyses with totals between 98.5 and 101.5 wt% were retained and are presented here.

**Table 2:** Representative silicate analyses in ferromagnesian chondrules in RBT 04133,8

Host														Clast					
Chondrule	Ch3	Ch3	Ch5	Ch5	Ch6	Ch6	Ch7	Ch7	Ch1^a^	Ch1^a^	Ch4	Ch4	Matrix	Ch21	Ch21	Ch22	Ch23	Ch23	Matrix
Chondrule Type	I	I	I	I	I	I	I	I	II	II	II	II		I	I	I	I	I	
Silicate Type	Ol	Px	Ol	Px	Ol	Px	Ol	Px	Ol	Ol-relict	Ol	Ol	Ol	Ol	Px	Ol	Ol	Px	Ol
Chemical composition (wt%)																			
Na_2_O	bdl	bdl	bdl	bdl	bdl	bdl	bdl	bdl	bdl	bdl	bdl	bdl	0.33	0.05	bdl	bdl	bdl	bdl	bdl
SiO_2_	41.79	58.79	42.06	57.36	40.30	58.07	41.46	58.50	35.63	41.78	34.35	34.52	31.16	42.11	58.75	34.10	41.94	58.69	33.12
MgO	53.48	38.55	54.21	37.78	47.05	35.81	52.96	38.50	25.88	54.19	21.37	20.96	17.85	55.23	38.70	21.74	54.47	39.40	17.70
Al_2_O_3_	0.08	0.93	bdl	1.57	bdl	0.43	bdl	0.90	0.05	0.07	bdl	bdl	1.97	bdl	1.02	bdl	bdl	0.53	bdl
P_2_O_5_	bdl	bdl	bdl	bdl	bdl	bdl	bdl	bdl	bdl	bdl	bdl	bdl	0.08	bdl	bdl	bdl	bdl	bdl	bdl
CaO	0.22	0.54	0.21	1.26	0.18	0.47	0.20	0.50	0.43	0.34	0.30	0.24	0.37	0.19	0.69	0.10	0.18	0.42	0.23
K_2_O	bdl	bdl	bdl	bdl	bdl	bdl	bdl	bdl	bdl	bdl	bdl	bdl	0.09	bdl	bdl	bdl	bdl	bdl	bdl
MnO	0.14	0.10	0.13	0.14	1.57	0.32	0.09	bdl	0.51	bdl	0.44	0.40	0.28	0.20	bdl	0.35	0.17	bdl	0.39
TiO_2_	bdl	0.15	bdl	0.33	bdl	0.08	bdl	0.22	0.04	0.04	bdl	bdl	0.10	bdl	0.19	bdl	bdl	0.08	bdl
FeO	4.43	1.37	3.67	0.51	11.09	3.75	5.30	0.82	37.52	3.71	42.61	43.69	42.69	2.50	0.54	42.91	3.68	0.67	47.63
Cr_2_O_3_	bdl	0.43	bdl	0.46	bdl	0.57	bdl	0.47	bdl	0.18	bdl	bdl	0.29	0.06	0.34	bdl	0.04	0.23	bdl
NiO	bdl	bdl	bdl	bdl	bdl	bdl	bdl	bdl	bdl	bdl	bdl	bdl	1.61	bdl	bdl	bdl	bdl	bdl	bdl
ZnO	bdl	bdl	bdl	bdl	bdl	bdl	bdl	bdl	bdl	bdl	bdl	bdl	bdl	bdl	bdl	bdl	bdl	bdl	bdl
Total	100.13	100.86	100.27	99.39	100.19	99.49	100.01	99.91	100.07	100.31	99.06	99.81	96.80	100.35	100.23	99.21	100.48	100.02	99.06
Cation formula based on 4 oxygens for olivine and 6 oxygens for pyroxene																			
Na	bdl	bdl	bdl	bdl	bdl	bdl	bdl	bdl	bdl	bdl	bdl	bdl	0.019	0.003	bdl	bdl	bdl	bdl	bdl
Si	0.998	1.968	0.999	0.998	1.991	1.991	0.995	1.971	1.002	0.993	1.004	1.005	0.951	0.996	1.971	0.996	0.995	1.973	0.996
Mg	1.904	1.924	1.920	1.737	1.831	1.831	1.896	1.934	1.085	1.921	0.931	0.909	0.812	1.947	1.935	0.947	1.927	1.975	0.793
Al	0.002	0.037	bdl	bdl	0.017	0.017	bdl	0.036	0.002	0.002	bdl	bdl	0.071	bdl	0.040	bdl	bdl	0.021	bdl
P	bdl	bdl	bdl	bdl	bdl	bdl	bdl	bdl	bdl	bdl	bdl	bdl	0.002	bdl	bdl	bdl	bdl	bdl	bdl
Ca	0.006	0.020	0.005	0.005	0.017	0.017	0.005	0.018	0.013	0.009	0.009	0.007	0.012	0.005	0.025	0.003	0.005	0.015	0.007
K	bdl	bdl	bdl	bdl	bdl	bdl	bdl	bdl	bdl	bdl	bdl	bdl	0.003	bdl	bdl	bdl	bdl	bdl	bdl
Mn	0.003	0.003	0.003	0.033	0.009	0.009	0.002	bdl	0.012	bdl	0.011	0.010	0.007	0.004	bdl	0.009	0.003	bdl	0.010
Ti	bdl	0.004	bdl	bdl	0.002	0.002	bdl	0.006	0.001	0.001	bdl	bdl	0.002	bdl	0.005	bdl	bdl	0.002	bdl
Fe	0.088	0.038	0.073	0.230	0.108	0.108	0.106	0.023	0.882	0.074	1.041	1.063	1.090	0.049	0.015	1.048	0.073	0.019	1.198
Cr	bdl	0.011	bdl	bdl	0.015	0.015	bdl	0.013	bdl	0.003	bdl	bdl	0.007	0.001	0.009	bdl	0.001	0.006	bdl
Ni	bdl	bdl	bdl	bdl	bdl	bdl	bdl	bdl	bdl	bdl	bdl	bdl	0.040	bdl	bdl	bdl	bdl	bdl	bdl
Zn	bdl	bdl	bdl	bdl	bdl	bdl	bdl	bdl	bdl	bdl	bdl	bdl	bdl	bdl	bdl	bdl	bdl	bdl	bdl
Total	3.001	4.004	3.001	3.002	3.991	3.991	3.005	3.999	2.997	3.003	2.996	2.995	3.016	3.005	4.000	3.004	3.004	4.011	3.004
Fe#	4.4	1.9	3.7	11.7	5.5	5.5	5.3	1.2	44.9	3.7	52.8	53.9	57.3	2.5	0.8	52.5	3.7	0.9	60.2
Mg#	95.6	97.1	96.3	88.3	93.6	93.6	94.7	97.9	55.1	96.3	47.2	46.1	42.7	97.5	98.0	47.5	96.3	98.3	39.8
Fe/Mg	0.0	0.0	0.0	0.1	0.1	0.1	0.1	0.0	0.8	0.0	1.1	1.2	1.3	0.0	0.0	1.1	0.0	0.0	1.5
Fe/Mn	32	14	29	7	12	12	55	–	73	–	96	109	149	12	–	120	22	–	121

a Relict grain-bearing type II chondrule.

bdl = below detection limit, Ch = chondrule, Ol = olivine, Ol-relict = relict grain-bearing olivine, Px = pyroxene.

Na, P, K, and Zn are bdl in all nonmatrix analyses.

Standards used for silicate analyses (with detection limits in wt%) were albite for Na (0.03); diopside for Si (0.03), Mg (0.03), and Ca (0.02); anorthite for Al (0.02); apatite for P (0.02); K-feldspar for K (0.02); rhodonite for Mn (0.04); rutile for Ti (0.02); fayalite for Fe (0.07); chromite for Cr (0.05); nickel metal for Ni (0.08); and zinc metal (sometimes gahnite) for Zn (0.13).

**Table 3:** Opaque mineral compositions (wt%) within the chondrules and matrix of the RBT 04133,8 host and clast

Host								Clast				
Ch/Mx	Ch5	Ch5	Ch5	Ch1	Ch1	Ch1	Ch1	Ch23	Ch23	Ch23	Mx1	Mx3
Chondrule Type	I	I	I	II	II	II	II	I	I	I	N/A	N/A
Opaque Type	Ni-rich metal	Ni-poor metal	Fe-sulfide	Ni-rich metal	Ni-poor metal	Fe-sulfide	Chromite	Ni-rich metal	Ni-poor metal	Fe-sulfide	Fe-sulfide	Ni-poor metal
Na	bdl	bdl	bdl	bdl	bdl	bdl	bdl	bdl	bdl	bdl	bdl	bdl
Si	bdl	0.04	0.03	0.03	bdl	0.05	0.16	0.05	bdl	0.03	bdl	bdl
Mg	bdl	bdl	bdl	bdl	bdl	bdl	2.28	bdl	bdl	bdl	bdl	bdl
Al	0.38	0.09	bdl	0.35	0.27	bdl	6.48	0.33	0.14	bdl	bdl	0.21
P	bdl	bdl	bdl	bdl	bdl	bdl	0.01	bdl	bdl	0.04	bdl	bdl
S	bdl	0.04	36.31	bdl	bdl	36.31	na	bdl	bdl	36.48	36.75	bdl
Ca	bdl	bdl	bdl	bdl	0.03	0.05	0.10	0.03	0.03	0.07	bdl	0.04
Cr	0.41	0.51	bdl	bdl	bdl	0.08	33.76	0.61	0.12	0.05	bdl	0.17
Mn	bdl	bdl	bdl	bdl	bdl	0.06	0.43	bdl	bdl	bdl	bdl	bdl
Ti	bdl	bdl	bdl	bdl	bdl	bdl	0.59	bdl	bdl	bdl	bdl	bdl
Fe	62.99	92.43	62.91	57.34	92.72	62.60	24.54	56.74	91.40	62.59	62.91	92.97
Ni	35.16	4.92	0.22	40.97	4.50	bdl	bdl	39.06	5.15	bdl	bdl	3.81
Co	0.24	1.08	bdl	0.35	1.52	bdl	na	0.48	2.76	bdl	bdl	1.72
Cu	bdl	bdl	bdl	0.28	bdl	bdl	na	0.20	bdl	bdl	bdl	bdl
Zn	bdl	bdl	bdl	bdl	bdl	bdl	bdl	bdl	bdl	bdl	bdl	bdl
V	na	na	na	na	na	na	0.48	na	na	na	na	na
O	na	na	na	na	na	na	30.85	na	na	na	na	na
Total	99.17	99.12	99.46	99.31	99.02	99.14	99.51	97.50	99.59	99.26	99.67	98.92

Ch = chondrule, Mx = matrix, na = not analyzed, bdl = below detection limits.

Na, Mg, P, Ti, Cu, and Zn all bdl for metal and sulfide data.

Standards used for metal and sulfide analyses were albite for Na (0.04); diopside for Si (0.02) and Mg (0.03); anorthite for Al (0.02) and Ca (0.02); indium phosphide for P (0.03); chalcopyrite for S (0.03); Fe (0.09), and Cu (0.13); chromium metal for Cr (0.03); manganese metal for Mn (0.05); titanium metal for Ti (0.03); nickel metal for Ni (0.10); cobalt metal for Co (0.09); gahnite for Zn (0.15); and vanadium metal for V (0.06).

O for spinel phases calculated by difference.

## Results

### Light Element Isotopic Compositions

**Fig. 1:**
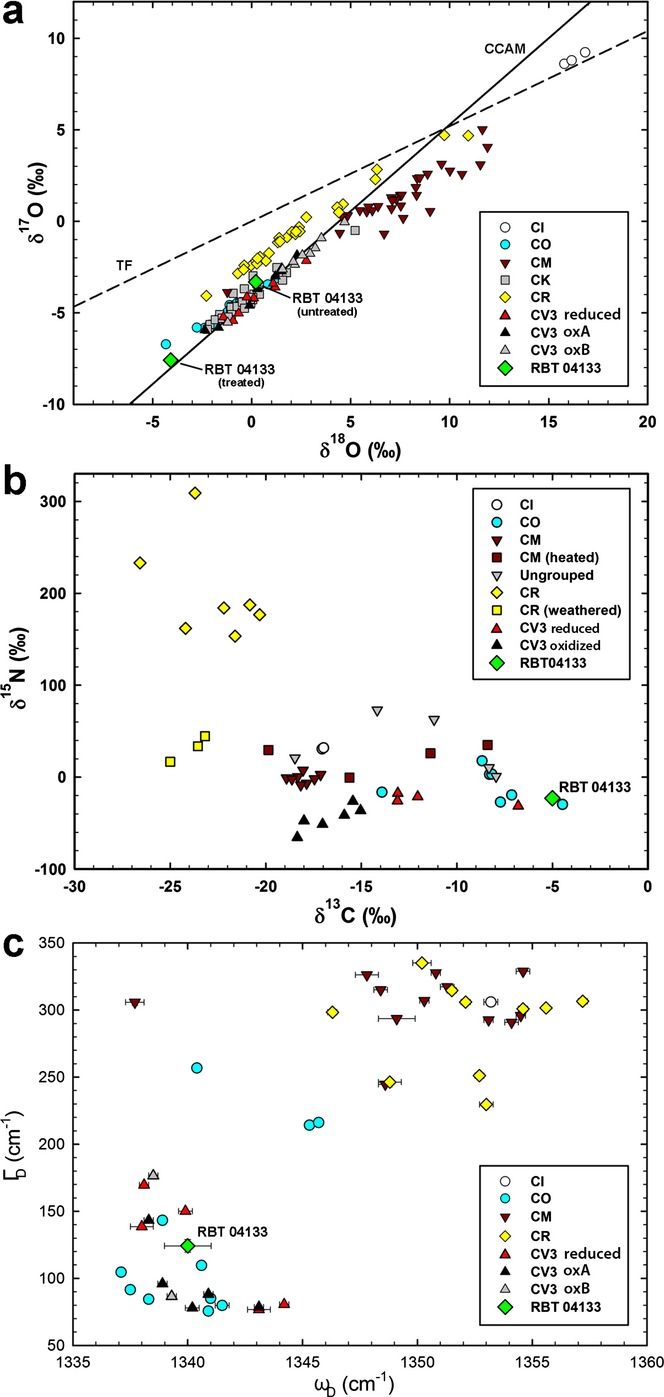
a) Whole-rock O-isotopic composition of RBT 04133,5 compared to CI, CO, CM, CK, CR, and CV chondrites; where TF = terrestrial fractionation line, and CCAM = carbonaceous chondrite anhydrous mineral line (δ^17^O = −4.17 + 0.945 × δ^18^O; Clayton et al. 1977). RBT 04133 plots with the CV3, CK, and CO chondrites, and away from the CR chondrites (which typically lie along their own mixing line above the CCAM line). All other data are from Weisberg et al. (1993), Clayton and Mayeda (1999), Martins et al. (2007), Choi et al. (2009), Greenwood et al. (2010), and Schrader et al. (2011). b) Carbon and N isotopic compositions of RBT 04133 IOM compared to those previously determined for IOM from other carbonaceous chondrites (Alexander et al. 2007). RBT 04133 is clearly not a CR chondrite (which exhibit isotopically heavier N and lighter C), but agrees with both CO and CV3_red_ chondrites. c) Raman D-band parameters (ω_D_ = position of D band, and Γ_D_ = full width at half maximum) for RBT 04133 IOM compared to IOM from other carbonaceous chondrites (Busemann et al. 2007). IOM from meteorites appear to cluster together within chondrite classes. RBT 04133 IOM clearly plots away from the CR chondrites, and within the same region as the CV and CO chondrites.

RBT 04133 has a whole-rock O-isotopic composition of δ^17^O = −3.32‰, δ^18^O = 0.21‰, and plots close to the carbonaceous chondrite anhydrous mineral (CCAM) line (e.g., Clayton et al. 1977) in the area occupied by analyses of CV, CK, and CO chondrites on a three-isotope plot (Fig.1a). As RBT 04133 is moderately weathered, an aliquot of powder was leached with HCl to remove weathering products (Greenwood et al. 2008) yielding a significantly lighter O-isotopic composition (δ^17^O = −7.59‰, δ^18^O = −4.09‰; Fig.1a).

RBT 04133 has whole-rock C and N isotopic compositions of δ^13^C = −12.2 ± 1.0‰ and δ^15^N = −23.5 ± 2.0‰. Carbon and N isotopic compositions of RBT 04133 IOM (δ^13^C = −5.0 ± 1.0‰, δ^15^N = −22.9 ± 2.0‰) agree with those of the CV3_red_ and CO chondrites (Fig.1b; Alexander et al. 2007). The mass fraction of C in the IOM residue is 69 wt%. The insoluble C content of the bulk meteorite is 0.6 wt%.

### MicroRaman Spectroscopy

Raman spectroscopy of RBT 04133 IOM generated both D- and G-band features (Fig.1c); ω_D_ = 1346.6 ± 1.0 cm^−1^, Г_D_ = 124.2 ± 4.7, ω_G_ = 1596.0 ± 1.3 cm^−1^, Г_G_ = 62.4 ± 2.1 (uncertainties are 1 standard deviation of the mean). These parameters indicate that the organic matter is moderately disordered and are comparable to previous studies of meteoritic IOM that has experienced moderate metamorphism in their parent bodies (Quirico et al. 2003; Bonal et al. 2006; Busemann et al. 2007). The D- and G-spectral bands yield a peak height ratio *I*_D_/*I*_G_ of 1.143 ± 0.028 that was used to infer a PMT of approximately 440 °C for RBT 04133 IOM (following the method of Busemann et al. 2007). The petrographic type of RBT 04133 lies between CV3_red_ Efremovka (3.1–3.4) and CV3_ox_ Grosnaja (approximately 3.6) on the basis of Г_D_, and between the CV3_ox_ chondrites Bali (>3.6) and Axtell (>3.6) on the basis of *I*_D_/*I*_G_ following the method of Bonal et al. (2006).

### Presolar Components

Analysis of RBT 04133 matrix revealed the presence of one presolar SiC (200 nm in diameter), one other C-anomalous grain (potentially presolar graphite or interstellar carbonaceous material; 250 nm in diameter), three presolar silicates (440–650 nm in diameter), and one presolar oxide (340 nm in diameter; Davidson et al. 2010). On the basis of its C and N isotopic compositions (δ^13^C = −711 ± 115‰, δ^15^N = −95 ± 70‰), the SiC grain is most likely a Y grain but could potentially be an X grain within error (Davidson et al. 2010). The Si isotopic composition of the SiC grain (determined during subsequent analyses) is solar within 3σ error (Davidson et al. 2010). The nature of the other C-anomalous grain (δ^13^C = −719 ± 132‰, δ^15^N below detection limit) could not be determined as it was sputtered away during analysis. However, as its isotopic composition falls outside the range of compositions previously reported for isotopically anomalous carbonaceous grains (e.g., Floss and Stadermann 2009a), it is more likely to have been presolar graphite. Two of the presolar silicate grains from RBT 04133 are ^17^O-enriched (δ^17^O = 270 ± 50‰, δ^18^O = −26 ± 18‰; δ^17^O = 163 ± 32‰, δ^18^O = −4 ± 14‰) and belong to the presolar oxide Group 1 (Nittler 1997), while the remaining two grains (one silicate, one oxide) are depleted in ^18^O (silicate: δ^17^O = −200 ± 80‰, δ^18^O = −166 ± 30‰; oxide: δ^17^O = −63 ± 99‰, δ^18^O = −160 ± 30‰) and from Group 3 (Nittler 1997). The matrix-normalized SiC abundance determined here from the matrix fragments (12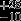 ppm) agrees with that determined by raster ion imaging of IOM within error (5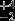 ppm; Davidson et al. 2014a). Errors associated with silicate (240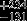 ppm) and oxide (33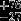 ppm) abundances determined here (based on counting statistics; Gehrels 1986; see Davidson et al. 2014a) are very large as a result of the low number of grains identified. As such, the apparently high abundances determined here are unreliable and would benefit from improved counting statistics by expanding the total amount of area analyzed. However, for this study, the important parameter is the presence of presolar silicates, which are typically only located in primitive chondrites (e.g., Nguyen et al. 2010).

### General Description, Mineralogy, and Petrography

Remnant fusion crust and several very large calcium-aluminum-rich inclusions (CAIs) are visible on the exterior of the whole RBT 04133 stone (approximately 1 cm in diameter; Fig. S1). It has experienced some weathering (B/C grade; Weisberg et al. 2008) that is evident from the Fe staining in the thin section (Fig. S2a).

**Fig. 2:**
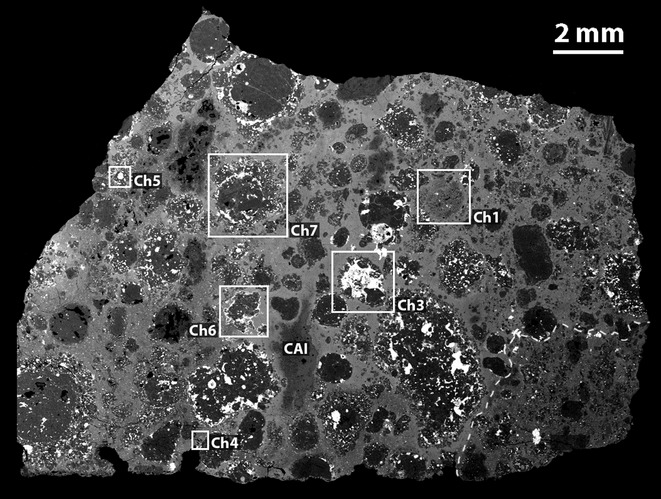
BSE image of the RBT 04133,8 thin section. Type I chondrules (e.g., Ch3, 5, 6, and 7) appear darker than type II chondrules (e.g., Ch1 and 4) as a result of their lower FeO-contents. Matrix appears bright, like type II chondrules, consistent with the fayalitic compositions of matrix olivine. The brightest regions are opaque mineral assemblages consisting primarily of sulfide and metal. The boundary between the clast and host material is indicated by a dashed line at the bottom right of the thin section. The clast is shown in higher resolution in Fig.3. Labels correspond to chondrules in Fig.4, opaque assemblages in Fig.5, and the CAI in Fig.8.

**Fig. 3:**
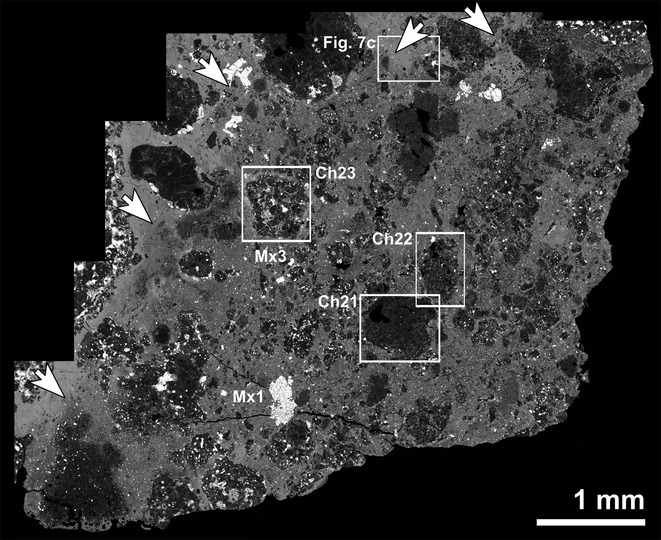
Photomosaic of 11 high-resolution BSE images showing the clast within the RBT 04133,8 thin section. The matrix in the clast region appears coarser-grained than the matrix in the host rock (arrows indicate the contact between the two regions; one area is shown in higher resolution in Fig.7c). Chondrule and matrix assemblage numbers correspond to those in Tables 2–4 and Figs. 4–6.

The RBT 04133,8 thin section consists of 95.6 vol% silicate and 4.4 vol% opaque minerals (Table 1). Silicate phases consist of 8.3 vol% refractory inclusions (CAIs and AOAs; ameboid olivine aggregates), 37.3 vol% chondrules (1.5 vol% Al-rich, 33.4 vol% type I [FeO-poor olivine; Fe/(Fe+Mg) < 10%, atomic ratio; e.g., Jones et al. 2005], and 2.4 vol% type II [FeO-rich olivine, Fe/(Fe+Mg) >10%, atomic ratio] chondrules), and 54.5 vol% matrix. The matrix/chondrule ratio is approximately 1.5. Opaque phases are primarily abundant sulfide (4.4 vol%), with lesser amounts of metal (0.4 vol%) and chromite (trace amounts), and terrestrial weathering products (1.7 vol%). No magnetite was observed. There is also a texturally distinct clast that constitutes 18.9 vol% of the thin section (Figs.2 and 3). A total of 45 whole chondrules within the host material (i.e., not clast) were found; their apparent diameters range from 0.02 to 3.41 mm with an average of 0.85 ± 0.69 mm (mean ± 1σ standard deviation).

#### Clast

**Fig. 4:**
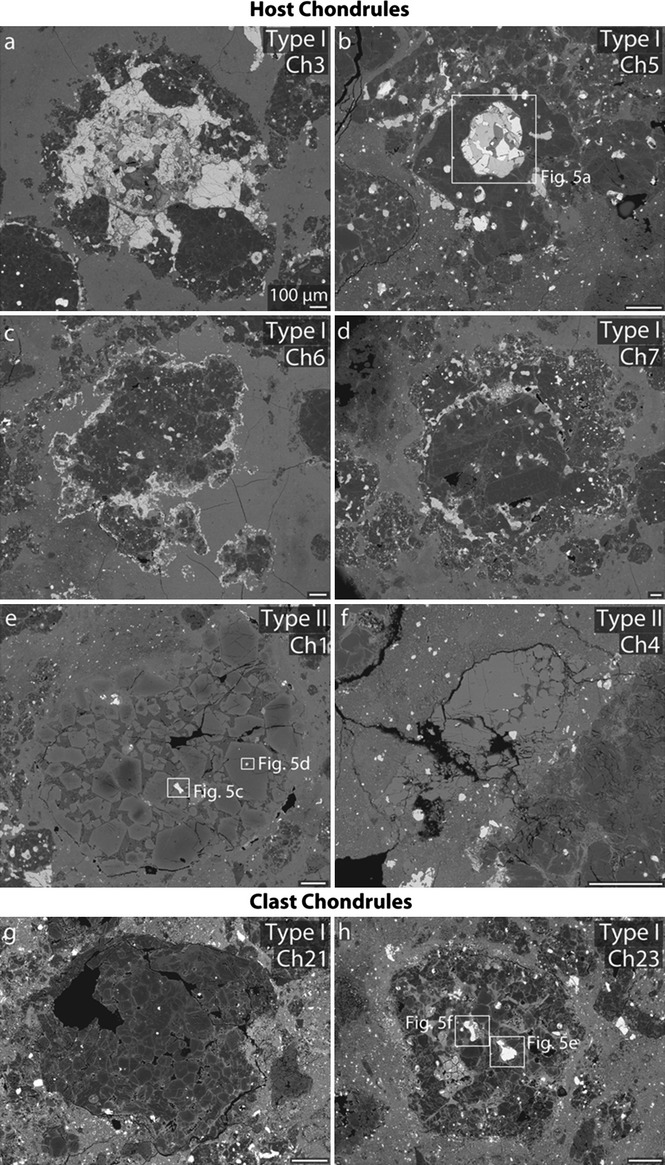
BSE images of representative chondrules in RBT 04133,8 (a–d) type I chondrules, and (e, f) type II chondrules. a) Opaque-rich chondrule 3, b) metal- and sulfide-rich chondrule 5, c) chondrule 6, d) compound chondrule 7, e), chromite- and relict grain-bearing chondrule 1, and f) relict-free chondrule 4. All chondrules have porphyritic textures. All scale bars are 100 μm. See Fig.2 for location of chondrules within thin section. Locations of opaque phases (Fig.5) are marked.

The texturally distinct clast contains smaller chondrules and coarser-grained matrix than the host (Fig.3). The clast appears much lighter than the host in plane-polarized light (Fig. S2a) as a result of the optical transparency of its coarser grained matrix. A total of 30 whole chondrules in the clast have apparent diameters ranging from 0.05 to 1.08 mm with an average of 0.31 ± 0.23 mm (mean ± 1σ standard deviation). Three type I chondrules in the clast were analyzed in detail, and as they were found to be compositionally indistinguishable from type I chondrules from the host (with the exception of the presence of Fe-rich rims around individual chondrule grains) their silicate compositions are not discussed separately here (Fig.4). There is no apparent evidence that the clast is a more terrestrially weathered part of the stone; opaque minerals in the clast's matrix do not appear to be more weathered than opaque minerals in the host matrix and the clast does not exhibit the Fe staining that is apparent in the more weathered areas of the section (Fig. S2a).

#### Chondrule Textural Types and Opaque Mineral Assemblages

The most dominant chondrule textural types are porphyritic-olivine-pyroxene (POP) and porphyritic-olivine (PO) chondrules (Fig.4). Type I chondrules (90 vol%) are more abundant than type II chondrules (6 vol%) and Al-rich chondrules (4 vol%). Of the 45 complete chondrules measured in the host material, five (11%) are also compound chondrules (e.g., chondrule 7; Fig.4d).

**Fig. 5:**
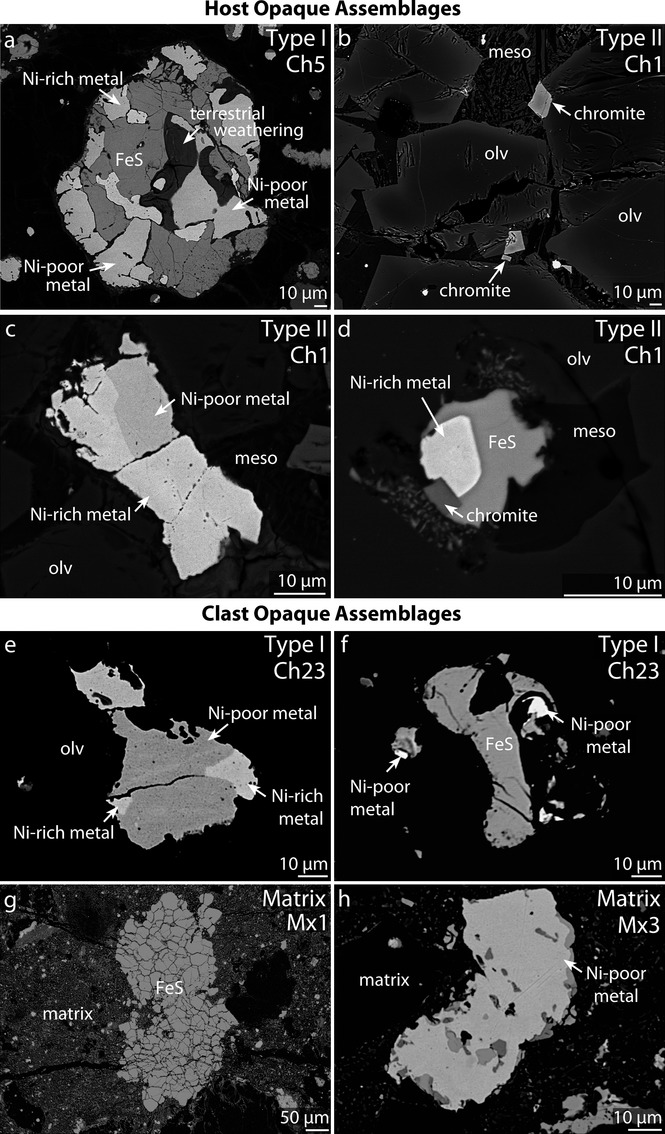
BSE images of representative opaque assemblages in RBT 04133,8 (a–d) host material, and (e–h) the clast. a) Sulfide-metal assemblage in chondrule 5 (type I), b) chromite grains in chondrule 1 (type II), c) Ni-rich and Ni-poor metal in chondrule 1 (type II), d) assemblage 3 in chondrule 1 (type II), e) Ni-rich and Ni-poor metal in chondrule 23 (type I), f) Fe-sulfide in chondrule 23, g) large Fe-sulfide in clast matrix (Mx1), and h) large metal grain in clast matrix (Mx3). See Fig.2 for location within thin section and Fig.4 for locations within silicates. meso = mesostasis, and olv = olivine.

**Fig. 6:**
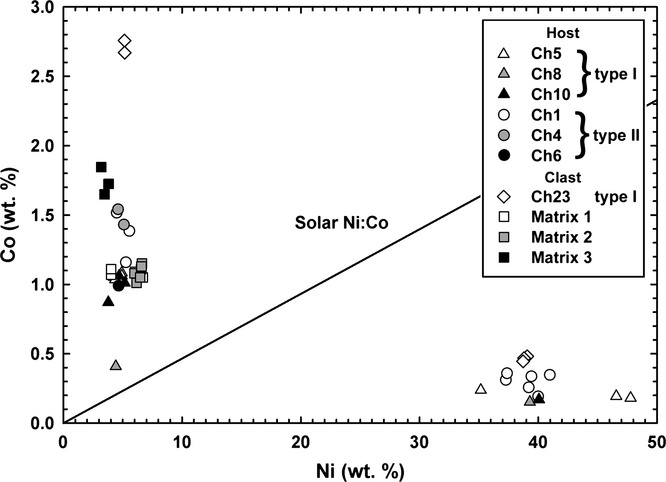
Cobalt and Ni (both wt%) content of individual metal grains in chondrules and matrix of RBT 04133,8. Nickel-rich metal is depleted in Co relative to Ni-poor metal, similar to observations of metal in thermally metamorphosed ordinary and CO chondrites (Kimura et al. 2008). The solar Ni:Co ratio line is shown for reference (data from Lodders et al. 2009).

Type I (FeO-rich; Fa_<10_) chondrules consist of both olivine and pyroxene phenocrysts (Table 2); olivine phenocrysts are either normally zoned or homogeneous with respect to Fa (Figs.4a–d). Overall, olivine in these chondrules has Fa_0.85–9.69_ (mean Fa_3.8±2.0_). These chondrules contain between 0.5 and 27.7 vol% opaque minerals (metals, sulfides, and chromite; Fig.5; Table 4). The opaque minerals are found throughout type I chondrules, but are typically concentrated along chondrule edges and are predominantly sulfides (see Fe-S-Ni composite X-ray map; Fig. S3b), although minor amounts of Fe,Ni metal are also present (Fig.5). When present, Fe,Ni metal in type I chondrules is either Ni-poor (≤7 wt% Ni; kamacite) or Ni-rich (20–50 wt% Ni; taenite). Ni-poor metal contains 91.6–93.8 wt% Fe, 3.8–5.2 wt% Ni, and 0.41–1.08 wt% Co. Ni-rich metal contains 35.2–47.8 wt% Fe, 35.2–47.8 wt% Ni, and 0.15–0.24 wt% Co (Table 3; Fig.6).

**Table 4:** Modal abundances, average olivine, and pyroxene compositions, and thermodynamic properties of individual chondrules within RBT 04133,8

			Olivine^b^		Pyroxene^c^						Modal abundances			Thermodynamic properties^d^				
Chondrule Number	Type	Texture^a^	Fa		Fs		En		Wo		Silicate	Rust + Chromite	Metal + Sulfide	a_Fa_	a_Fe_	IW	H_2_O/H_2_ at 1600 °C	Times solar H_2_O/H_2_ at 1600 °C
Host chondrules																		
1	II	PO	28.7	(14.6)							96.4	3.2	0.4	0.2134	0.78	−1.1	0.22	416
2	II	PO	25.8	(9.3)							98.8	1.2	0.0					
3	I	POP	6.3	(2.1)	3.1	(2.5)	95.7	(2.9)	1.2	(0.5)	64.5	7.8	27.7					
4	II	PO	53.8	(0.7)							96.9	0.9	2.3					
5	I	POP	4.5	(0.6)	1	(0.4)	97.8	(0.6)	1.2	(0.6)	60.3	22.9	16.8	0.0068	0.63	−2.4	0.05	91
6	II	POP	26.3	(20.7)	5.6	(2)	91.2	(1.7)	3.2	(1.5)	61.6	25.2	13.2	0.1405	0.91	−1.4	0.15	289
7	I	POP-comp.	4.3	(2.6)	0.9	(0.1)	98.1	(0.9)	0.2	(0.1)	83.3	16.1	0.5	0.0060	0.58	−2.4	0.05	94
10	I	POP-comp.	2.9	(1.1)	1.1	(0.2)	95.7	(0.3)	3.3	(0.1)	74.9	21.2	3.9	0.0028	0.85	−3.0	0.02	44
H	I	POP	2.8	(1.3)	2.4	(2.2)	96.1	(2.3)	1.5	(0.5)	82.6	13.2	4.2					
Clast chondrules																		
21	I	POP	3.9	(1.5)	0.8	*	98.0	*	1.3	*	75.3	24.1	0.6					
22	I	PO	5.0	(1.5)							74.6	24.2	1.2					
23	I	POP	5.4	(1.6)	1.1	(0.1)	98.1	(0.2)	0.9	(0.1)	77.8	18.2	4.0	0.0094	0.72	−2.4	0.05	94

a PO = porphyritic-olivine; POP = porphyritic-olivine pyroxene; POP-comp. = porphyritic-olivine pyroxene compound.

b Fa = fayalite number. Numbers in parentheses indicate one standard deviation of the mean (i.e., 1σ).

c Fs = ferrosilite number, En = enstatite number, Wo = wollastonite number. Numbers in parentheses indicate one standard deviation of the mean (i.e., 1σ).

d a_Fa_ = activity of Fe in olivine, a_Fe_ = activity of Fe in Fe,Ni metal, IW = iron-wüstite. Nb. Solar H_2_O/H_2_ ratio at 1600 °C = 5.28e^−4^ (Lodders et al. 2009).

* Indicates single analysis (therefore no standard deviation available).

Both Ni-poor and Ni-rich metal is present in a type I chondrule (Chondrule 23; Fig.6) within the clast. The Ni-poor metal contains 89.2–91.4 wt% Fe, 5.1 wt% Ni, and 2.67–2.76 wt% Co, while Ni-rich metal contains 56.6–56.9 wt% Fe, 38.7–39.1 wt% Ni, and 0.44–0.48 wt% Co (Table 3; Fig.6). The Ni-poor metal within Chondrule 23 contains higher Co-contents (up to 2.8 wt% Co) than metal in all of the host chondrules (up to 1.5 wt% Co; Fig.6).

Type II (FeO-rich; Fa_>10_) chondrules consist primarily of olivine phenocrysts, but also contain FeO-poor relict-grains (Figs.4e and 4f). Olivine phenocrysts in these chondrules have Fa_11.7–54.6_ (mean Fa_38.2±12.2_). Relict grains have Fa_3.7–9.3_ (mean Fa_6.0±2.4_). Type II chondrules typically contain fewer opaque minerals than type I chondrules (0–13 versus 1–17 vol%; Table 4). In contrast to what is seen in type I chondrules, the majority of opaque minerals are found in the interior of type II chondrules, and are mostly Fe,Ni metal (Figs.5c and 5d; Table 3), although sulfides and minor amounts of chromite (Fig.5d; Table 4) are also present. Chromite grains are subhedral to euhedral, internally homogeneous with an overall range in Cr_2_O_3_ content of 46.6–52.4 wt%, and located exclusively in type II chondrules (Figs.5b and 5d) (Davidson et al. 2009, 2011). Not all type II chondrules are chromite-bearing, although this may be an artifact of thin sectioning (e.g., Hezel 2007). When present, Fe,Ni metal in type II chondrules is either Ni-poor or Ni-rich. Ni-poor metal contains 91.6–92.7 wt% Fe, 4.5–5.6 wt% Ni, and 1.16–1.54 wt% Co. Ni-rich metal contains 57.3–60.0 wt% Fe, 37.3–41.0 wt% Ni, and 0.26–0.35 wt% Co (Table 3; Fig.6). Metal in type II chondrules generally contains more Co (up to 1.54 wt%) than metal in type I chondrules (up to 1.08 wt%) from the host material, but less than the Co-contents of metal in the clast (up to 2.8 wt% Co).

Nickel and Co are not positively correlated in Ni-rich and Ni-poor metal from individual type I (Ch5, Ch8, Ch10, and Ch23) and type II (Ch1) chondrules, i.e., the Ni/Co relationship is not solar (Fig.6). Sulfide minerals are either troilite (with atomic ratios of Fe/S = 1.00) or pyrrhotite (Fe/S from 0.98 to 1.02), with Ni contents below the detection limits in all but a few rare cases where Ni was present at up to 0.7 wt%. No pentlandite, (Fe,Ni)_9_S_8_, was seen.

#### Matrix

**Fig. 7:**
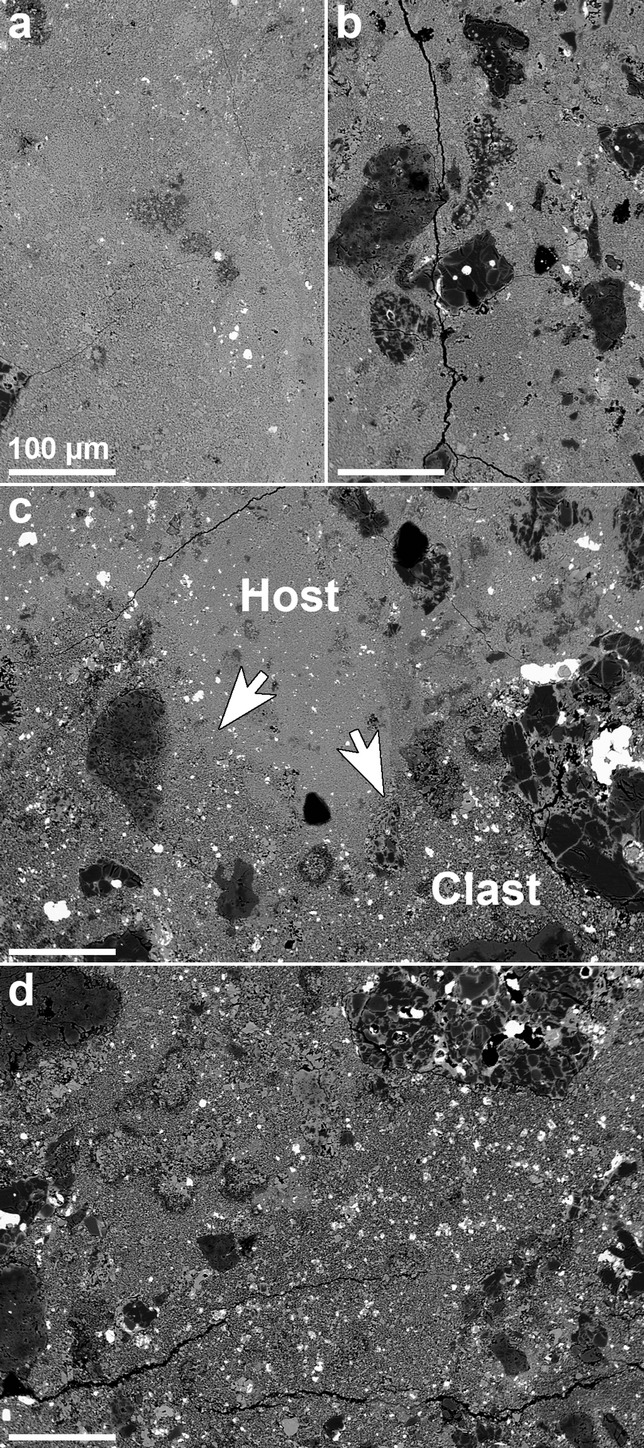
BSE images of a) fine-grained host matrix, b) abundant chondrules and chondrule fragments in fine-grained host matrix, c) the boundary between host and clast matrix (marked by white arrows), and d) coarse-grained clast matrix, which appears to contain abundant FeO-rich olivine grains and fewer chondrule fragments than the host matrix. All scale bars are 100 μm.

The host matrix consists of fine-grained silicates, metals, and sulfides. The host and clast matrices both contain abundant FeO-rich olivine grains with fayalite values of Fa_59_ and Fa_60_, respectively (Table 2). FeO-rich olivine grains are generally smaller in the host matrix (mostly <10 μm diameter; Fig.7a) than the clast matrix (many >10–20 μm diameter; Fig.7d). The largest opaque mineral phases (up to approximately 500 μm in diameter) within both matrices appear to be sulfides (e.g., Fig.5g). Fe,Ni metal in the clast matrix is Ni-poor and contains 90.2–93.0 wt% Fe, 3.2–6.7 wt% Ni, and 1.01–1.65 wt% Co (Table 3; Fig.6). Although they are compositionally similar, matrix textures and chondrule characteristics (e.g., average size) differ significantly between the clast and host material. The clast matrix is coarser grained than the host matrix (Fig.7) and contains abundant FeO-rich olivine grains and fewer chondrule fragments. All chondrules within the clast (all type I) possess thin, Fe-rich rims around individual grains (Figs.4g and 4h). The contact between the host and clast matrix is sharp (Fig.7).

#### Refractory Inclusions

**Fig. 8:**
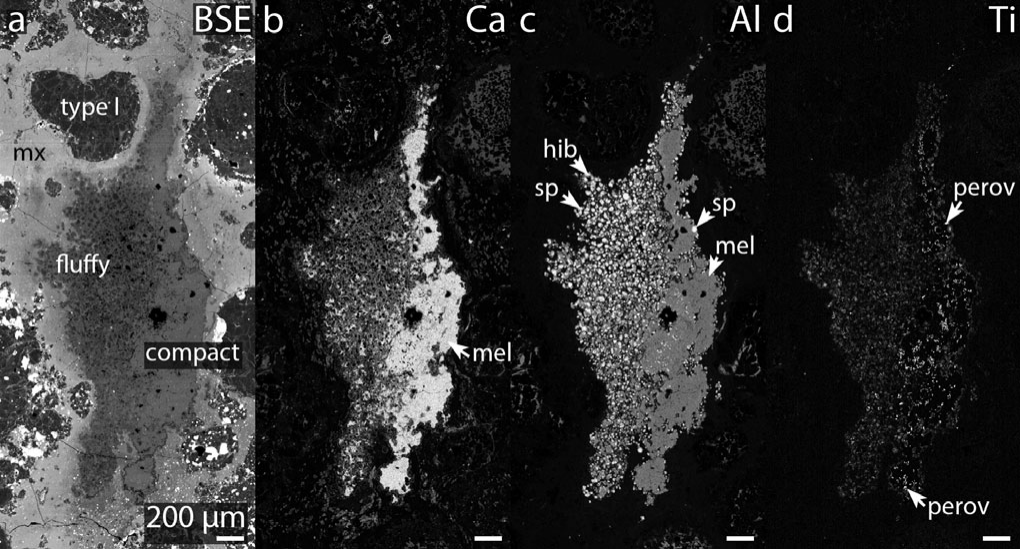
a) BSE image of a Ca-Al-rich inclusion in RBT 04133,8, and X-ray element maps of b) Ca, c) Al, and d) Ti. The CAI has both compact and fluffy textural characteristics. All scales the same. See Fig.2 and Fig. S3a for location in the thin section. mx = matrix, mel = melilite, hib = hibonite, sp = Al,Mg,Fe-spinel, and perov = perovskite.

The largest CAI present in the thin section (Figs.1 and 8; Fig. S3a) is petrographically and mineralogically consistent with being a Type A CAI (e.g., Brearley and Jones 1998). The CAI contains fine-grained (i.e., fluffy) and coarse-grained (i.e., compact) portions, and has apparent dimensions of 1.29 × 3.11 mm (Fig.8). The compact portion of the CAI mostly consists of melilite (Åk_1.3–3.1_), surrounding anhedral/euhedral perovskite grains (up to 20 μm in diameter), Al,Mg,Fe-spinel (70.0–71.0 wt% Al_2_O_3_, 23.4–27.3 wt% MgO, 0.2–6.0 wt% FeO), and minor amounts of hibonite (approximately 10 μm in diameter). The “fluffy” portion of this CAI consists of numerous nodules of spinel (approximately 20–50 μm in diameter) surrounded by melilite (Åk_0.7–3.5_), with minor amounts of hibonite (approximately 20–30 μm in diameter).

## Discussion

### RBT 04133: A Reduced CV3 Carbonaceous Chondrite

This study has employed a range of different analytical methods in order to determine the true classification of RBT 04133. All methods indicate that RBT 04133 is a CV3_red_ carbonaceous chondrite as is discussed further below.

The whole-rock O-isotopic composition of RBT 04133 indicates that it is not a CR chondrite, but is consistent with CO and CV3 chondrites (Fig.1a here; Clayton and Mayeda 1999; Greenwood et al. 2010). Terrestrial weathering is known to affect the O-isotopic composition of meteoritic samples (Greenwood and Franchi 2004). Acid-washing techniques have been used to “clean” meteorites, but they cannot readily discriminate between nebular/asteroidal and terrestrial alteration products and thus may also remove nonterrestrial phases. The O-isotopic compositions of such washed samples tend to shift away from the terrestrial fractionation (TF) line, with a co-variation in δ^18^O, the direction of which varies between Antarctic and hot desert weathering (Greenwood et al. 2012). However, the acid-leached RBT 04133 sample is much more ^16^O-rich than expected, yielding an O-isotopic composition more ^16^O-rich than any whole-rock CV3 reported by Greenwood et al. (2010). It is very unlikely that this can be explained by removal of terrestrial contamination alone and likely that a native ^16^O-poor component was lost too. Many CV3 chondrites contain magnetite (e.g., Howard et al. 2010), which is relatively ^16^O-poor (e.g., Davidson et al. 2014b). However, it seems unlikely that the leached component included magnetite as none was observed in the thin section and it should be more resistant to acid etching. While not conclusive, the highly ^16^O-rich composition indicates a stronger affinity with the CV rather than CR chondrites as the latter are inferred to have a relatively ^16^O-poor anhydrous silicate component (e.g., Clayton and Mayeda 1999; Schrader et al. 2011).

The C and N isotopic compositions of IOM from different meteorites cluster with others of the same class (Fig.1b) (Alexander et al. 2007), they also typically contain similar abundances of C in their IOM within a group. The C and N isotopic compositions of RBT 04133 IOM are consistent with data from both the CV3_red_ and the CO chondrites, and are significantly different from those of the CR chondrites and CV3_ox_ chondrites. The abundance of C in the IOM of RBT 04133 (69 wt%) is similar to those of the CRs, COs, and CV3_red_ (Alexander et al. 2007). The insoluble C content of the bulk meteorite (0.6 wt%) also agrees with other CV3_red_ chondrites (Alexander et al. 2007).

The IOM residue was also analyzed by Raman spectroscopy, which is useful for determining the relative degree of disorder (or maturity) of IOM (Fig.1c; e.g., Bonal et al. 2006; Busemann et al. 2007). Busemann et al. (2007) showed that, in terms of their spectral parameters, different meteorites cluster with those of the same class (Fig.1c). Considering this relationship, RBT 04133 does not appear to be a CR chondrite as its organics are not as primitive as those from known CR chondrites (Busemann et al. 2007). However, organic material in RBT 04133 is consistent with that in CV and CO chondrites.

Presolar grains were incorporated into all chondrite groups; SiC grains can be found in similar abundances in the most primitive members of each (e.g., Davidson et al. 2014a). Lower SiC abundances indicate destruction of grains/loss of their isotopic signatures as a result of parent body processing and thus indicate higher petrologic types. RBT 04133 was recently found to have a presolar SiC abundance (5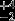 ppm; Davidson et al. 2014a) that is much lower than is expected for a CR chondrite (average 32 ± 9 ppm; Davidson et al. 2014a), but which agrees with the low abundance of approximately 0.48 ppm reported for the CV3_red_ chondrite Vigarano (Huss and Lewis 1995). This not only suggests that RBT 04133 is not a CR chondrite, it also indicates that this meteorite has experienced sufficient parent body processing to destroy the majority of the initial presolar grain inventory in its parent body.

The CV3 chondrites contain abundant matrix (40vol%), porphyritic ferromagnesian chondrules (45 vol%), refractory inclusions (10 vol%), and opaque mineral assemblages (0–5 vol%) (e.g., Weisberg et al. 2006; Rubin 2010; Jones 2012). These values are consistent with RBT 04133 being a member of the CV3 chondrite group. The average apparent diameter of full chondrules in RBT 04133 (0.85 ± 0.69 mm from 45 chondrules in the host) is consistent with the average chondrule diameter of approximately 1 mm for CV and CK chondrites, and larger than 0.15 mm for CO chondrites (Weisberg et al. 2006). The dominance of porphyritic chondrules over other chondrule types and the abundance of type I chondrules compared to type II chondrules are also consistent with CV chondrites (e.g., Weisberg et al. 2006; Rubin 2010; Jones 2012). Neither the abundance of refractory inclusions (Table 1) nor the size of individual CAIs in RBT 04133 (>1 mm diameter) are consistent with those in CR chondrites (CAIs in CR chondrites are typically <0.5 mm; Aléon et al. 2002). They are, however, similar to those commonly found in the CV and CK chondrites (Weisberg et al. 2006). It is unlikely that RBT 04133 is a CK chondrite as it lacks the characteristic abundant magnetite (e.g., Geiger and Bischoff 1995; Weisberg et al. 2006), and has matrix olivine compositions of Fa_57–60_ that are far more Fe-rich than those in CK chondrites (Fa_30_; Weisberg et al. 2006). On the basis of all petrographical and compositional data, RBT 04133 is a CV3_red_ chondrite. Furthermore, microstructural analysis of matrix from RBT 04133 has been found to be similar to other CV3_red_ chondrites (Abreu et al. 2013).

### Pre-Accretionary Formation Conditions

The compositions of mineral phases within chondrules provide information about the conditions under which they formed in the protoplanetary disk. The O fugacity (*f*O_2_) was calculated for each chondrule with the quartz-iron-fayalite buffer reaction using the average Fa content of olivine and the average Fe content of Fe,Ni metal, and was referenced to the iron-wüstite (IW) buffer (Table 4). We assume a silica activity of 0.9 for the three-component system metal-olivine-low-Ca pyroxene (e.g., Benedix et al. 2005). The online MELTS calculator (http://melts.ofm-research.org/CalcForms/index.html) of Sack and Ghiorso (1989) was used to obtain the activity of olivine. The necessary equilibrium constants were obtained from the HSC 7.0 Chemistry Reaction Equation module. The corresponding H_2_O/H_2_ ratios for all the chondrules were calculated assuming a temperature of 1600 °C, which is near the average of the temperature range in which chondrules likely crystallized (Hewins and Radomksy 1990). At 1600 °C, the type I chondrules studied here formed under H_2_O/H_2_ ratios from 0.02 to 0.05 (40–90 times the solar H_2_O/H_2_ ratio; IW–3.0 to IW–2.4), and the type II chondrules formed under H_2_O/H_2_ ratios from 0.15 to 0.22 (290–420 times the solar H_2_O/H_2_ ratio; IW–1.4 to IW–1.1). The *f*O_2_ values for type I chondrules in this study are similar to those calculated for the CV3_red_ chondrite Leoville (Zanda et al. 1994) (Table 4). Both type I and type II chondrules formed under H_2_O/H_2_ ratios enhanced relative to solar; which is consistent with those in other chondrite groups (e.g., Zanda et al. 1994; Lauretta et al. 2001; Schrader et al. 2013), but lower than those predicted by thermodynamic calculations for the high-temperature formation of FeO-rich olivine in the early solar system (e.g., Palme and Fegley 1990; Wood and Hashimoto 1993; Ebel and Grossman 2000).

The oxidizing conditions present during chondrule formation may also be recorded in the relationships between Ni versus Cr and Ni versus P in the metal of RBT 04133 (Table 3). The depletion of Cr and P in metal is similar to the results of high-temperature oxidation and sulfidation experiments of Fe,Ni alloys (Schrader and Lauretta 2010). Depletion of Cr and P in metal in unequilibrated chondrites (including the CV3_red_ Leoville) has been attributed to the presence of micron-sized chromite and phosphate inclusions within the metal that form by oxidation during chondrule cooling and also thermal metamorphism (Zanda et al. 1994). However, unlike chondrules from the CR chondrites (Schrader et al. 2013), the abundance of sulfides in chondrules in RBT 04133 does not increase with increasing oxidation state (i.e., sulfides are abundant in type I chondrules while they are uncommon in type II chondrules). This suggests either that (1) the sulfides are secondary (i.e., parent body), or (2) they are pre-accretionary and the oxidation state and sulfidation state of chondrules in the CV chondrites were decoupled (i.e., H_2_O/H_2_ was decoupled from H_2_S/H_2_ during chondrule formation). If the sulfides are secondary, they should be present in similar relative abundances within both type I and type II chondrules; however, this is not the case (Figs.4a–d versus 4e and 4f). In addition, if the sulfides formed via parent body aqueous alteration, the presence of additional secondary minerals, such as phyllosilicates and magnetite, would be expected. Marrocchi and Libourel (2013) reported the presence of abundant sulfides within type I chondrules in the CV3_red_ chondrite Vigarano, and based on the apparent co-crystallization of troilite and low-Ca pyroxene during high-temperature events they suggest that sulfides formed during chondrule formation. Furthermore, Hezel et al. (2010) argue that the bulk chondrule Fe isotope compositions of chondrules from oxidized CV chondrites Mokoia, Allende, and Grosnaja are indicative of evaporation and re-condensation during chondrule formation; implying that metal and sulfides in these chondrules are pre-accretionary. We suggest that sulfides within type I chondrules from RBT 04133 are also pre-accretionary, providing further evidence that some sulfide minerals formed prior to accretion of the CV chondrite parent body. This contradicts the general belief that CV chondrite sulfides formed by parent body aqueous alteration (e.g., see Brearley (2006) review and discussion therein).

### Parent Body Processing

There is no apparent evidence for significant aqueous alteration within the RBT 04133 host or clast, attested to by the lack of abundant alteration minerals (e.g., phyllosilicates and magnetite). However, there do appear to be several indicators of thermal metamorphism. Nickel-rich metal in RBT 04133 is depleted in Co relative to Ni-poor metal (Fig.6); this is similar to the composition of metal in the CV3_red_ chondrites Efremovka (Nazarov et al. 2000) and Vigarano (Krot et al. 2000) that both have estimated subtypes of 3.1–3.4 (Bonal et al. 2006; Busemann et al. 2007). Similar observations have been made for the thermally metamorphosed ordinary and CO chondrites (Kimura et al. 2008) and the shock heated CR chondrite GRA 06100 (Abreu and Bullock 2013). More pristine (i.e., less-altered) chondrites, such as the majority of the CR chondrites (e.g., Weisberg et al. 1993), exhibit a positive relationship between Ni and Co indicating that Co becomes mobile during thermal metamorphism. The Ni and Co content of metal in RBT 04133 is most similar to that in type 3.5–3.9 LL chondrites and the CO chondrite Y-791717, which is considered to be of either petrologic type 3.3 (Busemann et al. 2007) or 3.6 (Kimura et al. 2008). This suggests that RBT 04133 is likely of metamorphic type 3.3 or greater. Furthermore, the Co content of Ni-poor metal in the clast is significantly greater than that of Ni-poor metal in the host material indicating that clast metal has experienced higher degrees of thermal metamorphism than the host metal. However, as the chondrule silicates are unequilibrated (Fa_0.9–54.6_), this thermal metamorphism was likely mild. As RBT 04133 is of petrologic type >3.2, it is not possible to use the Cr_2_O_3_ content of FeO-rich olivine to determine its subtype (Grossman and Brearley 2005). Furthermore, this method has not been calibrated for CV3 chondrites.

Thermal metamorphism has also been shown to alter chromite textures in type II chondrules from euhedral to subhedral (Johnson and Prinz 1991). The composition (high Cr_2_O_3_; 46.6–52.4 wt%) and morphology (euhedral/subhedral) of chromite within type II chondrules of RBT 04133 are also consistent with mild thermal metamorphism (Figs.5b and 5d and Table 3) (Davidson et al. 2011).

Lower presolar SiC abundances indicate that presolar grains have been destroyed by parent body processing and thus indicate higher petrologic types (e.g., Davidson et al. 2014a). RBT 04133 was found to have presolar SiC abundances (12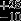 in matrix, this work, and 5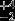 ppm in IOM; Davidson et al. 2014a) that are lower than is expected for a CR chondrite (average 32 ± 9 ppm; Davidson et al. 2014a), suggesting that RBT 04133 is not a CR chondrite and that it has experienced sufficient parent body processing to destroy the majority of its initial presolar SiC grain inventory in its parent body. Presolar SiC grains are typically more resistant to low temperature parent body aqueous alteration than presolar silicates (Zinner 2014). For example, presolar SiC grains in the CR chondrites are present in similar abundance in even the most aqueously altered CR1 chondrite GRO 95577 (Davidson et al. 2014a). However, presolar silicates have been mostly destroyed in the heavily aqueously altered CR2 chondrite Renazzo (no presolar silicate grains were found in one NanoSIMS ion imaging study (Floss and Stadermann 2005), and only two were found in another (Leitner et al. 2012)). Although highly speculative given the low number of grains identified, a rough presolar silicate/oxide ratio of 3 indicates that this sample has undergone significant parent body alteration (e.g., Floss and Stadermann 2009b). It is curious that presolar silicates are still present in RBT 04133 when many SiC grains appear to have either been destroyed (unlikely at a PMT of 440 °C) or have lost their isotopic signatures via diffusion. Another study identified only a single presolar silicate grain and no SiC grains in the thermally metamorphosed CO3 chondrite QUE 97416 (Bose et al. 2014). These observations indicate that the progressive destruction or modification of presolar SiC grains begins before presolar silicate grains are completely unidentifiable.

The inferred PMT of 440 °C for RBT 04133 IOM determined by microRaman spectroscopy of IOM is intermediate between two other CV3_red_ chondrites, Vigarano (CV3.1–3.4; PMT = 330–370 °C; Bonal et al. 2006; Busemann et al. 2007) and MET 01017 (CV3.7; PMT = 580–590 °C; Busemann et al. 2007). This agrees with petrographic observations that suggest RBT 04133 has experienced mild thermal metamorphism. A PMT of 440 °C would be sufficient to destroy the majority, but not all, of the presolar grains incorporated into the RBT 04133 parent body prior to metamorphism (e.g., Davidson et al. 2014a).

The source of heat for thermal metamorphism of RBT 04133 on the CV parent body is likely to be either radiogenic or low-velocity impact heating. Linear foliation of chondrules has been observed in Leoville (CV3_red_) and attributed to impact heating (Scott et al. 1992; Rubin 2012). However, foliation has been experimentally shown to only occur in CV chondrites at impact pressures >10 GPa (e.g., Nakamura et al. 1995, 2000). Linear foliation of chondrules was not observed in RBT 04133. While the presence of a distinct CV3_red_ clast (Figs.2 and 3) shows that RBT 04133 is brecciated and thus experienced impact processing, RBT 04133 did not experience high velocity impacts that could lead to extensive thermal metamorphism; consistent with compositional and textural indications for mild thermal alteration.

Clasts of oxidized CV material (CV3_oxA_ and CV3_oxB_) have been identified in Vigarano (CV3_red_; Krot et al. 2000), and Mokoia (CV3_ox_) consists of both oxidized lithologies (Krot et al. 1998). Although the clast present in RBT 04133 consists of CV3_red_ material like the host rock, the coarser-grained matrix, Fe-rich rims on olivine, and compositions of metal suggest that it is more thermally metamorphosed. This further demonstrates the brecciated nature of the CV parent body.

## Summary and Implications

We have performed a multitechnique characterization of the Antarctic meteorite RBT 04133. Data presented here conflict with its initial classification as a CR2 (Weisberg et al. 2008). Petrographically, RBT 04133 appears to be a CV3_red_, based on the presence of large CAIs and chondrules, the apparent lack of magnetite, and a matrix composition of Fa_59–60_. This is in agreement with its whole-rock C, N, and O-isotope compositions, and the Raman spectral characteristics of its IOM.

Thermodynamic calculations indicate that type I and type II chondrules in RBT 04133 formed under different, but relatively oxidizing conditions (H_2_O/H_2_ ratios = 40–420 times solar). This is also reflected in the Ni, Cr, and P compositions of their metal, which are similar to those of other CV3_red_ chondrites. Sulfide minerals are abundant in type I chondrules of RBT 04133, but are uncommon in type II chondrules. We suggest that they formed prior to accretion of the CV chondrite parent body.

There is no evidence that suggests RBT 04133 experienced significant aqueous alteration. However, its low presolar grain abundances, the PMT of 440 °C estimated from Raman spectral data, the metal compositions, the chromite compositions and morphologies, and the presence of unequilibrated silicates indicate that RBT 04133 is mildly thermally altered in nature and appears to be of petrologic type ≥CV3.3_red_. The presence of a more thermally metamorphosed clast of reduced CV3 material within RBT 04133 indicates that it, like several other CV chondrites, is a breccia.

## Editorial Handling

Dr. Christine Floss

## SUPPORTING INFORMATION

**Fig S1:** Full RBT 04133 stone prior to cutting. Large Ca-, Al-rich inclusions (white areas) are present on a macroscale on the exterior of the stone (almost 1 cm in diameter). Areas of fusion crust (dark patches) are also visible. Scale cube is 1 cm. Image courtesy of NASA JSC.

**Fig S2:** Full thin section maps of RBT 04133,8 shown in (a) plane-polarized light (PPL), and (b) cross polarized light (CPL). The clast is optically transparent in PPL as a result of its coarser-grained matrix than the host. Matrix in the host material is generally dark in PPL except for areas where Fe staining can be seen (as a result of terrestrial weathering). Both maps are shown at the same scale.

**Fig S3:** Composite RGB (red-green-blue) X-ray elemental maps of the RBT 04133,8 thin section. a) MgCaAl (Mg: red, Ca: green, and Al: blue) showing the presence of a large CAI (blue), and b) FeSNi (Fe: red, S: green, and Ni: blue) demonstrating the abundance of sulfide minerals (yellow) in and around chondrules. Both maps are shown at the same scale.
