# Supplementary figures and images for: Petrography, stable isotope compositions, microRaman spectroscopy, and presolar components of Roberts Massif 04133: A reduced CV3 carbonaceous chondrite
Source: Meteorit Planet Sci. 2014 Nov 7;49(12):2133–51. doi: 10.1111/maps.12377 (PMC4657624; doi:10.1111/maps.12377)

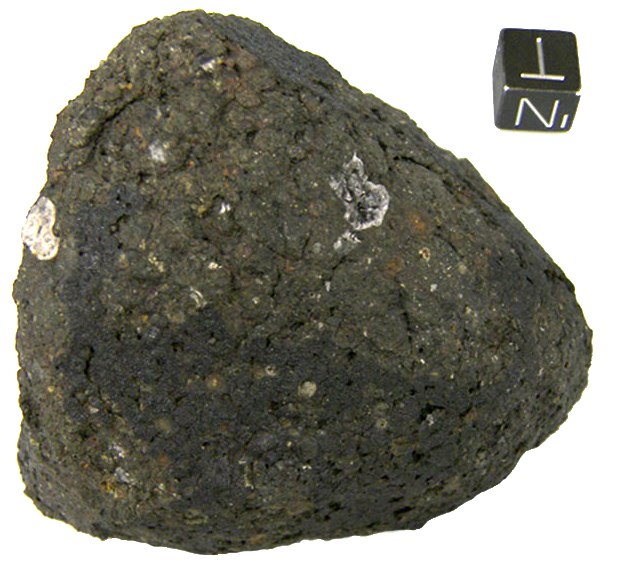

Supplement: Supplementary file 1 — Fig S1: Full RBT 04133 stone prior to cutting. Large Ca-, Al-rich inclusions (white areas) are present on a macroscale on the exterior of the stone (almost 1 cm in diameter). Areas of fusion crust (dark patches) are also visible. Scale cube is 1 cm. Image courtesy of NASA JSC. [file maps0049-2133-sd1.tif]

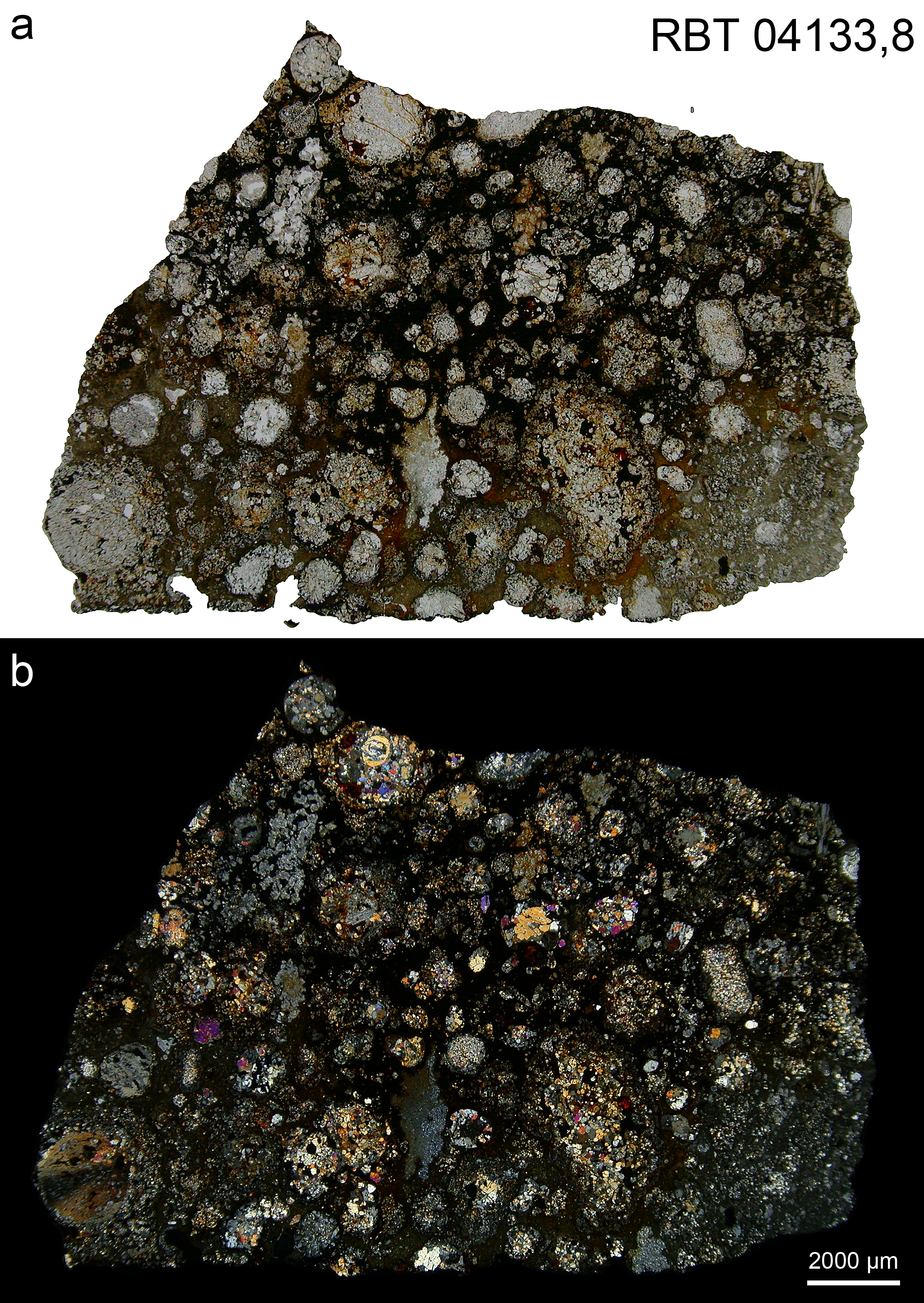

Supplement: Supplementary file 2 — Fig S2: Full thin section maps of RBT 04133,8 shown in (a) plane-polarized light (PPL), and (b) cross polarized light (CPL). The clast is optically transparent in PPL as a result of its coarser-grained matrix than the host. Matrix in the host material is generally dark in PPL except for areas where Fe staining can be seen (as a result of terrestrial weathering). Both maps are shown at the same scale. [file maps0049-2133-sd2.tif]

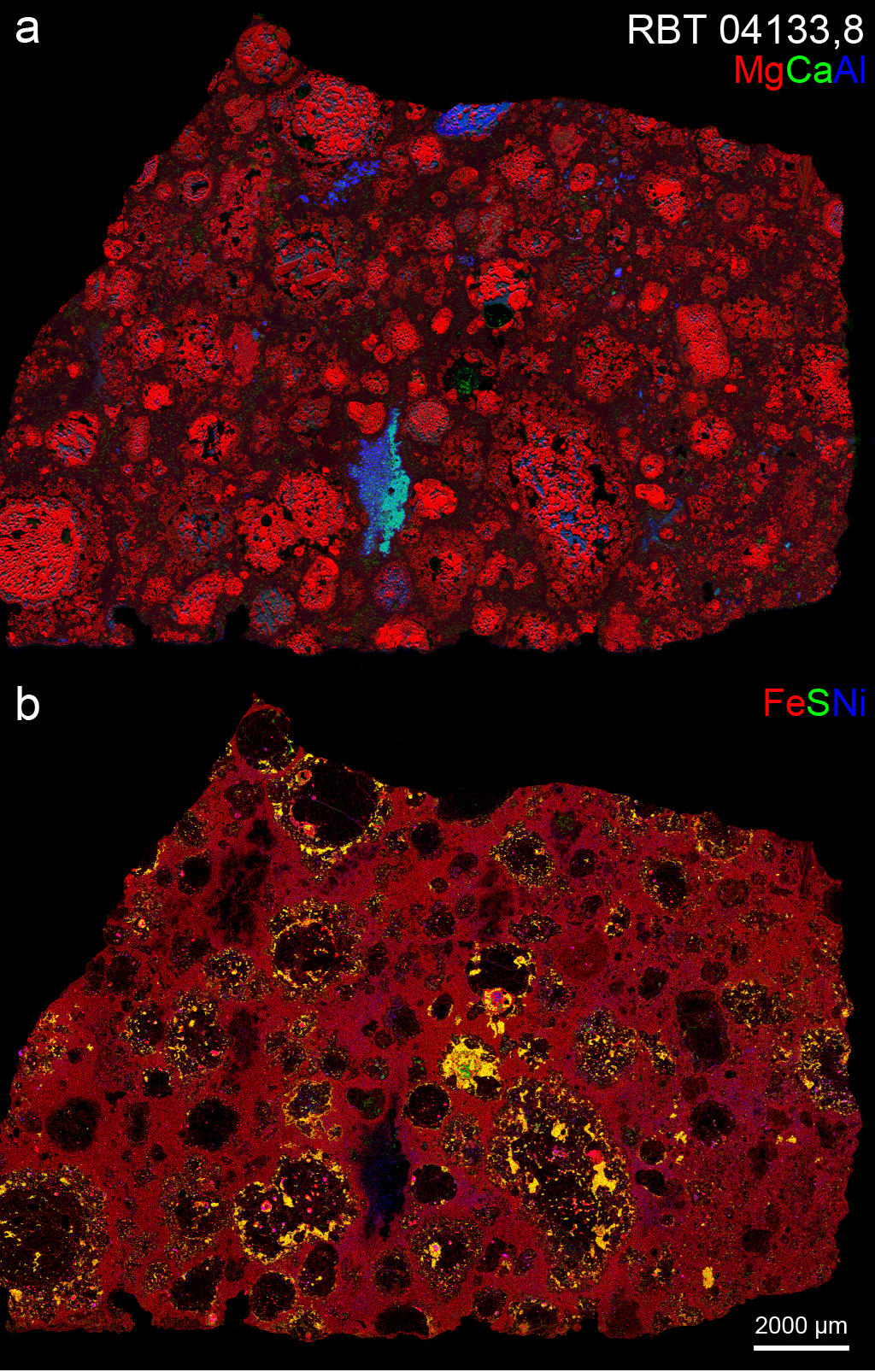

Supplement: Supplementary file 3 — Fig S3: Composite RGB (red-green-blue) X-ray elemental maps of the RBT 04133,8 thin section. a) MgCaAl (Mg: red, Ca: green, and Al: blue) showing the presence of a large CAI (blue), and b) FeSNi (Fe: red, S: green, and Ni: blue) demonstrating the abundance of sulfide minerals (yellow) in and around chondrules. Both maps are shown at the same scale. [file maps0049-2133-sd3.tif]
